# Prevalence and factors associated with pentavalent vaccination: a cross-sectional study in Southern China

**DOI:** 10.1186/s40249-023-01134-8

**Published:** 2023-09-15

**Authors:** Jianing Xu, Yujie Cui, Chuican Huang, Yuanyuan Dong, Yunting Zhang, Lichun Fan, Guohong Li, Fan Jiang

**Affiliations:** 1 School of Public Health, Shanghai Jiao Tong University School of Medicine, 227 South Chong Qing Road, Shanghai, 200025 China; 2 China Hospital Development Institute, Shanghai Jiao Tong University, Shanghai, China; 3 Department of Child Health Care, Hainan Women and Children’s Medical Center, Haikou, China; 4 Child Health Advocacy Institute, National Children’s Medical Center, Shanghai Children’s Medical Center, School of Medicine, Shanghai Jiao Tong University, Shanghai, China; 5 Department of Developmental and Behavioral Pediatrics, National Children’s Medical Center, Shanghai Children’s Medical Center, School of Medicine, Shanghai Jiao Tong University, 227 South Chong Qing Road, Shanghai, 200025 China; 6 Pediatric Translational Medicine Institute, National Children’s Medical Center, Shanghai Children’s Medical Center, School of Medicine, Shanghai Jiao Tong University, Shanghai, China; 7 Shanghai Center for Brain Science and Brain-Inspired Technology, Shanghai, China

**Keywords:** Early child development, Pentavalent, Diphtheria-tetanus-acellular pertussis inactivated poliomyelitis and *Haemophilus influenzae* type B vaccine, Vaccination coverage, Influencing factor, China

## Abstract

**Background:**

Immunization is one of the most far-reaching and cost-effective strategies for promoting good health and saving lives. A complex immunization schedule, however, may be burdensome to parents and lead to reduced vaccine compliance and completion. Thus, it is critical to develop combination vaccines to reduce the number of injections and simplify the immunization schedule. This study aimed to investigate the current status of the pentavalent diphtheria-tetanus-acellular pertussis inactivated poliomyelitis and *Haemophilus influenzae* type B conjugate (DTaP-IPV/Hib) vaccination in Southern China as well as explore the factors in the general population associated with uptake and the differences between urban and rural populations.

**Methods:**

A cross-sectional study was conducted with recently enrolled kindergarten students in Hainan Province between December 2022 and January 2023. The study employed a stratified multistage cluster random sampling method. Information regarding the demographic characteristics and factors that influence decisions were collected from the caregivers of children via an online questionnaire. Multivariate logistic regression was used to determine the factors associated with the status of DTap-IPV/Hib vaccinations.

**Results:**

Of the 4818 valid responses, 95.3% of children were aged 3–4 years, and 2856 (59.3%) held rural *hukou*. Coverage rates of the DTaP-IPV/Hib vaccine, from 1 to 4 doses, were 24.4%, 20.7%, 18.5%, and 16.0%, respectively. Caregivers who are concerned about vaccine efficacy [adjusted odds ratio (a*OR*) = 1.53, 95% confidence interval (*CI*): 1.30–1.79], the manufacturer (a*OR* = 2.05, 95% *CI*: 1.69–2.49), and a simple immunization schedule (a*OR* = 1.26, 95% *CI*: 1.04–1.54) are factors associated with a higher likelihood of vaccinating children against DTaP-IPV/Hib. In addition, caregivers in urban areas showed more concern about the vaccine price (*P* = 0.010) and immunization schedule (*P* = 0.022) in regard to vaccinating children.

**Conclusions:**

The DTaP-IPV/Hib vaccine coverage rate in Hainan Province remains low. Factors such as lower socioeconomic status, cultural beliefs, concerns about vaccine safety, and cost may hinder caregivers from vaccinating their children. Further measures, such as health education campaigns to raise knowledge and awareness, and encouragement of domestic vaccine innovation, which would reduce out-of-pocket costs, could be implemented to improve the coverage of DTap-IPV/Hib vaccination.

**Graphical Abstract:**

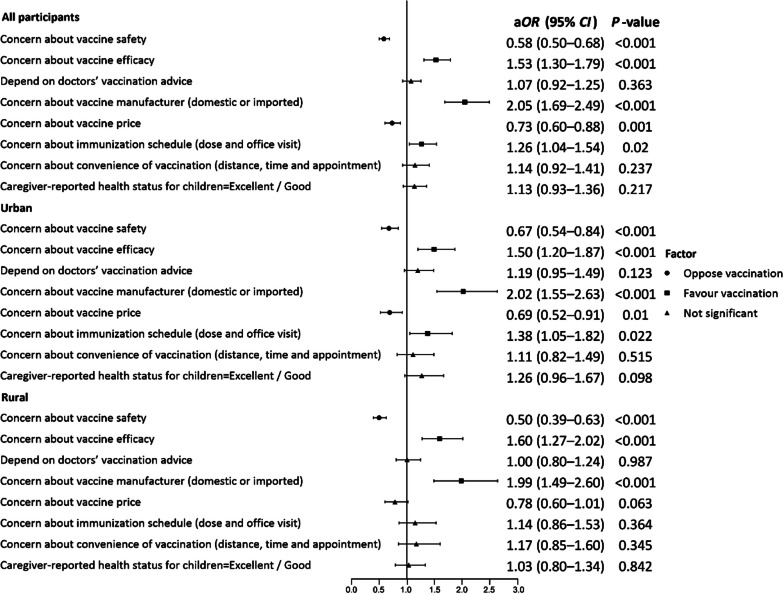

**Supplementary Information:**

The online version contains supplementary material available at 10.1186/s40249-023-01134-8.

## Background

Early child development (ECD) is an essential element of the Sustainable Development Goals and serves as the foundation of adult health and well-being [1]. Although substantive progress has been made in tackling under-5 mortality, with a reduction in the number of childhood deaths from 5.9 million in 2015 to 5 million in 2021, many children who survive are not able to thrive due to the threat of infectious diseases [2]. Among the five major areas of ECD, the area of health emphasizes childhood immunization. Vaccines annually prevent 2–3 million fatalities and safeguard millions more from disease and disability [3]. As one of the most far-reaching health interventions, immunization is an incredibly cost-effective strategy for promoting good health and saving lives. A study in 94 low- and middle-income countries estimated that every US dollar (USD) 1 invested in immunization generates a return of USD 51.8 in broader societal benefits of people who live longer and healthier lives [4].

The World Health Organization (WHO) developed Immunization Agenda 2030 to reduce mortality and morbidity from vaccine-preventable diseases (VPDs) from the period of 2021 to 2030 [5]. The current National Immunization Program (NIP) in China provides, at no cost, vaccines for eligible-aged children to prevent 12 VPDs and reduce by 99% the incidence of these diseases [6]. In addition, the number of recommended vaccines during childhood has increased significantly. Currently, children can receive ~ 20 injections by the age of 2 years to complete their immunization schedule, although the number of injections may increase in the coming years due to an increasing number of new diseases and vaccines. Evidence suggests, however, that a complex immunization schedule may be burdensome to parents and healthcare providers and can even lead to reduced vaccine compliance and completion [7, 8].

To address these concerns, many international organizations recommend that countries develop combination vaccines, which can be produced by grouping multiple antigens into one injection [9, 10]. In 2010, the Chinese National Medical Products Administration approved the pentavalent diphtheria-tetanus-acellular pertussis inactivated poliomyelitis and *Haemophilus influenzae* type B conjugate vaccine (DTaP-IPV/Hib) (Pentaxim^®^, Sanofi Pasteur Limited, Marcy l’Etoile, France), which can prevent more than five high incidences of morbidity and mortality diseases [11]. Until now, it was the highest degree of combination vaccines available in China. Numerous studies have demonstrated the good immunogenicity and safety profile of the DTaP-IPV/Hib combination vaccine, which is equal to the separately administered vaccine components [12, 13]. The DTaP-IPV/Hib combination vaccine offers a safe and effective alternative for reducing the number of injections from 12 to 4, which can reduce pain and discomfort and prevent potential side effects for children, save time and money, and reduce the loss of productivity for caregivers [14, 15]. Notably, China, as the sole WHO member country that has not incorporated the Hib vaccine into its NIP, exhibits a relatively low national coverage rate of only 33%, thereby experiencing a significant residual burden of Hib disease [16]. The use of the DTaP-IPV/Hib combination vaccine could contribute to enhancing Hib coverage, which allows for better and wider protection against infectious diseases and decreases the cost of disease management [17].

The DTap-IPV/Hib vaccination rates in many developed countries are far higher and were 94.4% in England and over 93.7% in Canada [18, 19]. The vaccine is imported, optional, and self-paid (Category 2), and the DTap-IPV/Hib vaccination rate in China significantly varies across different regions, but the overall rates are low. According to previous studies, the DTap-IPV/Hib vaccine coverage exhibited variations, with rates that range from 18.51% in Hangzhou during 2017 to 6.28% in Chongqing during 2015 [20, 21]. Considering the financial responsibility of caregivers in China to fully cover the cost of the DTap-IPV/Hib vaccine through out-of-pocket payments, it becomes imperative to assess the actual immunization status and the factors that influence DTap-IPV/Hib vaccine uptake within the country. Nevertheless, it is unfortunate that there is a lack of comprehensive information available about the utilization of the DTap-IPV/Hib vaccine and the factors that influence its uptake within the Chinese context.

In 2018, the Chinese government made the strategic decision to establish Hainan Province as the nation’s inaugural free trade port, operating under the socialist system. To facilitate the importation of pharmaceuticals and sanitary equipment, Hainan Free Trade Port (HFTP) has implemented a range of convenient and preferential laws and policies. This development is expected to enhance the accessibility of imported vaccines for local residents [22]. Hence, this study aimed to investigate the current status of DTap-IPV/Hib vaccination in Hainan Province as an example and explore the potential influencing factors in the general population as well as the differences between urban and rural populations. The study also sought to provide recommendations for increasing the vaccination rate, including tailored preparation to address hesitancy, and build vaccine literacy in China.

## Methods

### Study design and ethic

This study is part of a larger cross-sectional survey on the intervention strategies for ECD, which includes, among others, immunization, responsive caregiving, and early learning. The survey was conducted with a population of newly enrolled kindergarteners in Hainan Province from December 12, 2022, to January 8, 2023. Although it is mandatory for all 3-year-old children who reside in Hainan Province to enroll in kindergarten, there may be variations in the actual age at which they are enrolled (95% of the children were 3–4 years old, but there were a few children who were 2 or 5 years old). Ethics approval was obtained from the Research Ethics Board of the Hainan Women and Children’s Medical Center (2020-002). This paper includes data only from vaccination surveys and uses components of the cross-sectional questionnaire relevant to the aims of this paper.

### Study participants and randomization

Newly enrolled kindergarteners in Hainan Province in 2022 were recruited, and those who had foreign nationality or studied in special education schools were excluded. A stratified multistage cluster random sampling approach was employed for the cross-sectional study. First, primary sampling units (PSUs) were set at the county-level administrative region. There are a total of 3 groups and 24 categories of PSUs, including 8 municipal districts, 6 county-level cities, and 10 counties/autonomous counties. Half of the units in each group were randomly selected.

**Fig. 1:**
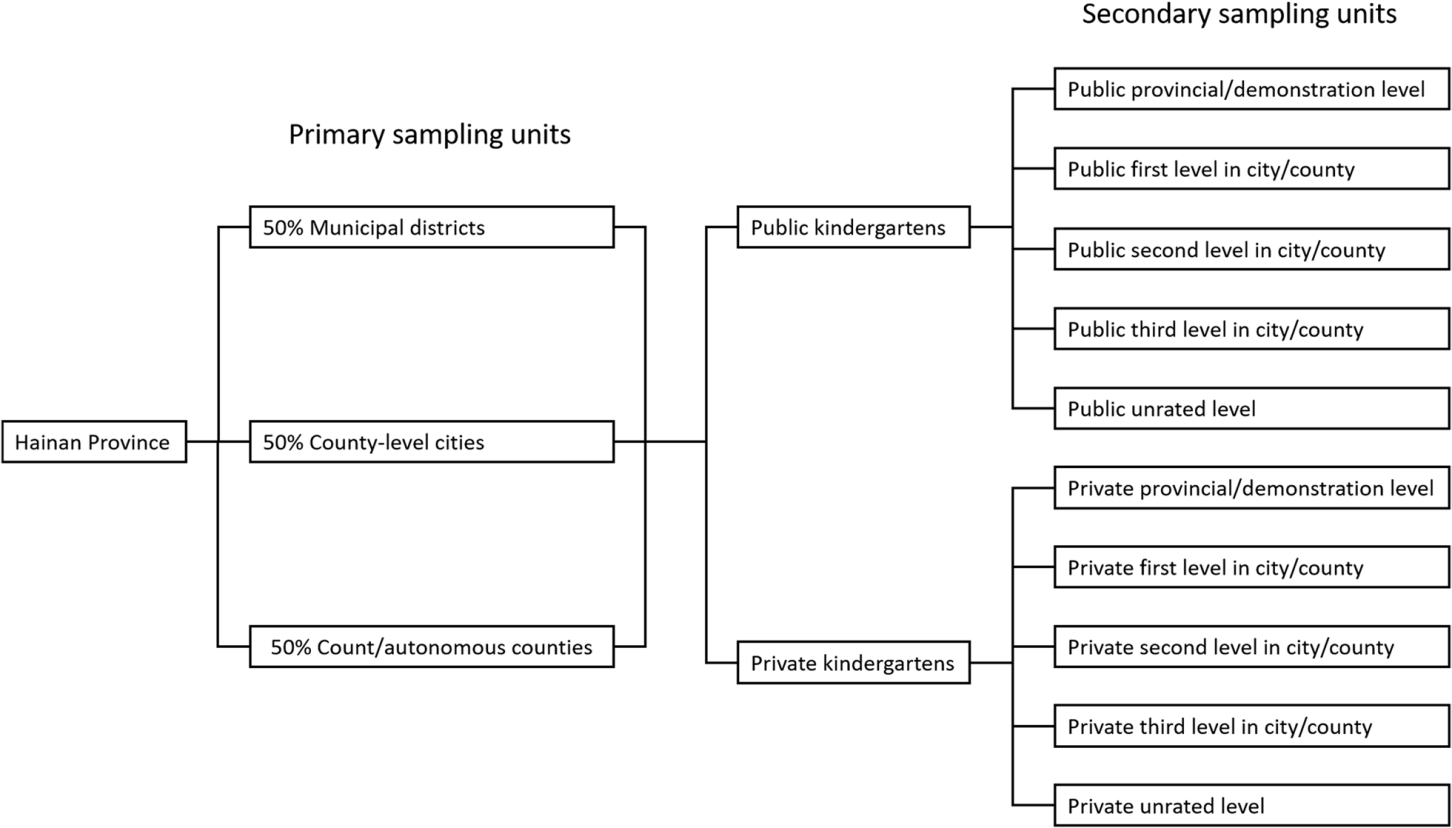
Stratified sample units of kindergartens of different levels in Hainan. There are 4 prefecture-level cities, 5 county-level cities, and 10 counties/autonomous counties in the Hainan administrative division. Among the 4 prefecture-level cities, there are 4 municipal districts each in Haikou and Sanya Cities. Danzhou City is taken as a county-level city, as it governs only streets and towns. We deleted Sansha City due to underpopulation. Thus, there is a total of 3 groups and 24 categories of primary sampling units, including 8 municipal districts, 6 county-level cities, and 10 counties/autonomous counties

We then defined secondary sampling units (SSUs) based on the kindergarten’s ownership (public or private) and level (provincial/demonstration level; first, second, or third level in city/county; and unrated level). There are a total of 120 categories of SSUs. In each SSU, one or two kindergartens were randomly selected, and all the enrolled children in the junior grade were invited to participate in the survey (Fig. 1). Random sampling was conducted, using a list of random numbers, by an individual epidemiologist who was not involved in any other research activities of this survey. A total of 8478 children from 180 kindergartens were randomly selected as participants. All caregivers of children who participated in the study were informed about the intention of the study and gave their electronic informed consent at the beginning of the online survey.

### Sample size and power analysis

The sample size was calculated with the following formula based on an error *α* = 0.05, $Z_{1 - \alpha /2}$ = 1.96, and allowance error δ = 0.02: $n = \left( {\frac{{Z_{1 - \alpha /2} }}{\delta }} \right)^{2} \times p \times \left( {1 - p} \right)$. Based on the whole ECD study, we adopted the early Human Capability Index to more comprehensively assess the development of children. In accordance with our preliminary pilot study, the estimated risk of poor development was found to be 18%, which is slightly lower than the average rate of 20%, observed within the Chinese population [23]. Assuming a conservative estimate of 20% for the risks of poor development in Hainan, we determined that the calculated sample size required was 1537. After estimating 70% valid data, the total number of participants was expected to be 2196.

### Data collection and quality control

Based on a review of the literature, we developed a structured questionnaire to collect data on demographic characteristics and factors that influence the choices of the DTaP-IPV/Hib vaccine. We implemented expert consultation to ensure the scientific validity and rationale of the questionnaire. We then conducted a pilot study with a random sample of 128 caregivers in two kindergartens to ensure the comprehension of the questionnaire. After the pilot study, a few modifications were made to ensure that the questions were comprehensible and interpreted as intended. The results of the pilot study were not included in the main study.

The data collection was carried out by the Maternal and Children Health Care System in Hainan Province, China. At the beginning of the survey, we provided standard training for the head of each PSU, who then provided training and guidance to the kindergartens within their jurisdiction to ensure that they carried out this survey, following the uniform process. The kindergarten representatives were responsible for checking the children’s personal information and guiding parents or caregivers to finish the online survey within two weeks.

In addition, a web-based questionnaire and research management platform were set up. The selected kindergartens were requested to upload the properly formatted information about their children (including name, gender, kindergarten, class, and date of birth) to the questionnaire platform, and the questionnaire platform generated a unique login code for each child [24]. Both the link to the research and the unique login code were distributed to parents through the Maternal and Children Health Care System and kindergarten teachers. The questionnaire collection process is strictly quality controlled by various levels of regulatory systems. Using the login code, all parents accessed the online questionnaire to double-check the child’s personal information and gave informed consent to participate in the survey.

After collecting questionnaires, we excluded those with missing important and obvious logical errors. Valid data with complete basic information and DTaP-IPV/Hib vaccination status were included in the analysis.

### Measures

Using the researcher-designed questionnaire, we obtained the general demographic characteristics of the participants, including children’s age, gender, *hukou* (the location of registered residency of the child), and ethnic group; administrative division, rank, and type of kindergartens; premature delivery, basic medical insurance, commercial medical insurance, number of children in the family, and previous vaccination status in NIP; caregivers’ relationship with the children, education level, and employment status; and annual household income.

Acceptance of vaccination is an outcome behavior that results from a complex decision-making process that can be potentially influenced by a wide range of factors. As caregivers play a key role in vaccination, we also assessed their influence using the “3Cs” model, which was first proposed to the WHO EURO Vaccine Communications Working Group in 2011. The “3Cs” model is a professionally validated theoretical framework for vaccination determinants, comprised of confidence, convenience, and complacency factors [25]. We designed eight questions that were incorporated into the 3Cs in our study. To make subsequent analysis clearer, we categorized responses into two categories: “Yes” or “No.”

### Statistical analysis

All variables were categorical and represented as frequencies with percentages. The characteristics of participants who had previously been vaccinated for DTaP-IPV/Hib and those who had not were compared using a chi-square test.

The relationship between the explanatory variables (demographic characteristics of caregivers and children) and the outcome variable (vaccinating their children against DTaP-IPV/Hib) was examined by multivariate logistic regression. The outcome variable was dichotomized into “Vaccinated” (at least 1 dose) and “Unvaccinated.” An adjusted odds ratio (a*OR*) with a 95% confidence interval (*CI*) for each variable were calculated.

A comprehensive non-responder analysis was conducted. The available data from the Hainan Women and Children’s Medical Center system were used to conduct an analysis of non-response to evaluate whether the non-responders differed systematically from the responders of the survey. Then, a subgroup analysis was performed, which examined differences in the variables among the groups. All statistics were managed by Microsoft Excel version 2010 (Microsoft Corporation, Redmond, WA, USA) and analyzed using SPSS version 24.0 (SPSS Inc., Chicago, IL, USA). Two-sided *P*-values < 0.05 were considered significant.

## Results

### Demographic characteristics of respondents

**Fig. 2:**
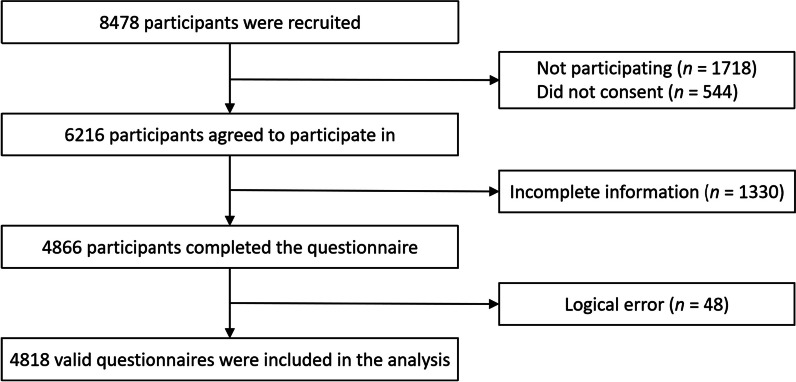
Flowchart of sample selection

A total of 4818 valid questionnaires were analyzed in this study, for a valid response rate of 56.8% (Fig. 2). Of the 4818 responses, most were aged ≤ 3 years (75.2%), the majority were from the Han population (80.4%), and 2856 (59.3%) held rural *hukou*. Almost two-thirds of the families had more than one child (66.1%), and the vast majority (92.5%) of children completed the immunization program of Hainan Province at the target age. Among the respondents, mothers predominated (75.3%), 70.0% of the caregivers were employed, and 31.6% had a 4-year college or associate’s degree. With regard to non-NIP vaccine determinants, the safety (51.0%) and efficacy (44.1%) of the vaccine are the two core issues with which caregivers have always been concerned, and more than one-third (39.3%) of caregivers depend heavily on doctors’ vaccination advice. Details are provided in Table 1.

**Table 1:** DTaP-IPV/Hib vaccination status among respondents by characteristics

Variable	Total (*N* = 4818)	Unvaccinated (*n* = 3644)	Vaccinated (*n* = 1174)	*P-*value
Children’s characteristics				
Gender				0.852
Male	2642 (54.8)	2001 (54.9)	641 (54.6)	
Female	2176 (45.2)	1643 (45.1)	533 (45.4)	
Age (years)				0.136
≤ 3	3623 (75.2)	2721 (74.7)	902 (76.8)	
4–5	1195 (24.8)	923 (25.3)	272 (23.2)	
* Hukou*^a^				< 0.001
Urban	1962 (40.7)	1262 (34.6)	700 (59.6)	
Rural	2856 (59.3)	2382 (65.4)	474 (40.4)	
Ethnic group				< 0.001
Han	3874 (80.4)	2837 (77.9)	1037 (88.3)	
Minority	944 (19.6)	807 (22.1)	137 (11.7)	
County-level administrative region				< 0.001
Municipal district	1882 (39.1)	1218 (33.4)	664 (56.6)	
County-level city	956 (19.8)	726 (19.9)	230 (19.6)	
County/autonomous county	1980 (41.1)	1700 (46.7)	280 (23.9)	
Rank of kindergarten				< 0.001
Provincial/demonstration level	1649 (34.2)	1110 (30.5)	539 (45.9)	
City-county level	2376 (49.3)	1934 (53.1)	442 (37.6)	
Unrated level	793 (16.5)	600 (16.5)	193 (16.4)	
Type of kindergarten				0.001
Public	3425 (71.1)	2547 (69.9)	878 (74.8)	
Private	1393 (28.9)	1097 (30.1)	296 (25.2)	
Premature delivery				0.03
Yes	476 (9.9)	364 (10.0)	112 (9.5)	
No	4157 (86.3)	3125 (85.8)	1032 (87.9)	
Undisclosed	185 (3.8)	155 (4.3)	30 (2.6)	
Basic medical insurance				< 0.001
Yes	4308 (89.4)	3203 (87.9)	1105 (94.1)	
No	245 (5.1)	211 (5.8)	34 (2.9)	
Undisclosed	265 (5.5)	230 (6.3)	35 (3.0)	
Commercial medical insurance				< 0.001
Yes	1116 (23.2)	700 (19.2)	416 (35.4)	
No	3121 (64.8)	2471 (67.8)	650 (55.4)	
Undisclosed	581 (12.1)	473 (13.0)	108 (9.2)	
Only child in the family				< 0.001
Yes	1635 (33.9)	1161 (31.9)	474 (40.4)	
No	3183 (66.1)	2483 (68.1)	700 (59.6)	
Complete vaccination for target-age children in immunization program				< 0.001
Yes	4459 (92.5)	3331 (91.4)	1128 (96.1)	
No	359 (7.5)	313 (8.6)	46 (3.9)	
Caregivers’ characteristics				
Primary caregiver				< 0.001
Mother	3629 (75.3)	2805 (77.0)	824 (70.2)	
Father	391 (8.1)	292 (8.0)	99 (8.4)	
Other	798 (16.6)	547 (15.0)	251 (21.4)	
Education level				< 0.001
Middle school and below	1963 (40.7)	1655 (45.4)	308 (26.2)	
Senior high school/technical school	1088 (22.6)	825 (22.6)	263 (22.4)	
College/associate’s degree	1524 (31.6)	997 (27.4)	527 (44.9)	
Bachelor’s degree and above	66 (1.4)	21 (0.6)	45 (3.8)	
Undisclosed	177 (3.7)	146 (4.0)	31 (2.6)	
Employment status				< 0.001
Employed	3373 (70.0)	2449 (67.2)	924 (78.7)	
Unemployed	1148 (23.8)	959 (26.3)	189 (16.1)	
Undisclosed	297 (6.2)	236 (6.5)	61 (5.2)	
Annual household income (CNY)				< 0.001
< 30,000	787 (16.3)	670 (18.4)	117 (10.0)	
30,000–49,999	480 (10.0)	402 (11.0)	78 (6.6)	
50,000–99,999	504 (10.5)	373 (10.2)	131 (11.2)	
100,000–299,999	814 (16.9)	521 (14.3)	293 (25.0)	
≥ 300,000	141 (2.9)	57 (1.6)	84 (7.2)	
Undisclosed	2092 (43.4)	1621 (44.5)	471 (40.1)	
Confidence				
Concern about vaccine safety				0.958
Yes	2455 (51.0)	1856 (50.9)	599 (51.0)	
No	2363 (49.0)	1788 (49.1)	575 (49.0)	
Concern about vaccine efficacy				< 0.001
Yes	2125 (44.1)	1441 (39.5)	684 (58.3)	
No	2693 (55.9)	2203 (60.5)	490 (41.7)	
Depend on doctors’ vaccination advice				0.164
Yes	1893 (39.3)	1452 (39.8)	441 (37.6)	
No	2925 (60.7)	2192 (60.2)	733 (62.4)	
Concern about vaccine manufacturer (domestic or imported)				< 0.001
Yes	907 (18.8)	558 (15.3)	349 (29.7)	
No	3911 (81.2)	3086 (84.7)	825 (70.3)	
Convenience				
Concern about vaccine price				0.002
Yes	1170 (24.3)	925 (25.4)	245 (20.9)	
No	3648 (75.7)	2719 (74.6)	929 (79.1)	
Concern about immunization schedule (dose and office visit)				< 0.001
Yes	957 (19.9)	644 (17.7)	313 (26.7)	
No	3861 (80.1)	3000 (82.3)	861 (73.3)	
Concern about convenience of vaccination (distance, time, and appointment)				< 0.001
Yes	719 (14.9)	489 (13.4)	230 (19.6)	
No	4099 (85.1)	3155 (86.6)	944 (80.4)	
Complacency				
Caregiver-reported health status for children				0.001
Excellent/good	3868 (80.3)	2886 (79.2)	982 (83.6)	
Fair/poor	950 (19.7)	758 (20.8)	192 (16.4)	

Values are shown as *n* (%). *P*-values were derived from Chi-square tests

*CNY* Chinese Yuan

^a^*Hukou* represents the location of registered residency of the child (urban or rural)

### Factors associated with DTaP-IPV/Hib vaccination

The immunization coverage rates of the DTaP-IPV/Hib vaccine, from 1 to 4 doses, were 24.4%, 20.7%, 18.5%, and 16.0%, respectively, in Hainan Province. In the multivariate regression model, DTaP-IPV/Hib vaccination status differed significantly in terms of the children’s *hukou* (*P* < 0.001), and ethnic group (*P* < 0.001); administrative division (*P* < 0.001) and rank of kindergartens (*P* < 0.001); basic medical insurance (*P* < 0.004), commercial medical insurance (*P* < 0.001), and previous vaccination status in NIP (*P* < 0.001); caregivers’ education level (*P* < 0.001) and employment status (*P* < 0.045); and annual household income (*P* < 0.003). We then adjusted all socioeconomic and demographic characteristics of respondents. Caregivers who feel positively toward vaccine efficacy (a*OR* = 1.53, 95% *CI*: 1.30–1.79), the manufacturer (a*OR* = 2.05, 95% *CI*: 1.69–2.49), and immunization schedule (a*OR* = 1.26, 95% *CI*: 1.04–1.54) are more likely to vaccinate their children against DTaP-IPV/Hib. Those who are more concerned about vaccine safety (a*OR* = 0.58, 95% *CI*: 0.50–0.68) and price (a*OR* = 0.73, 95% *CI*: 0.60–0.88) are less likely to vaccinate their children against DTaP-IPV/Hib (Table 2).

**Table 2:** Multivariate analysis of the factors that influence DTaP-IPV/Hib vaccination (*N* = 4818)

Variable	Model 1		Model 2^a^	
	a*OR* (95% *CI*)	*P-*value	a*OR* (95% *CI*)	*P-*value
Children’s characteristics				
* Hukou*^b^ (Ref: Rural)	1.46 (1.24–1.72)	< 0.001	1.42 (1.20–1.67)	< 0.001
Ethnic group (Ref: Minority)	1.48 (1.19–1.84)	< 0.001	1.49 (1.19–1.86)	< 0.001
County-level administrative region (Ref: County/autonomous county)		< 0.001		< 0.001
Municipal district	1.96 (1.64–2.34)	< 0.001	1.95 (1.63–2.34)	< 0.001
County-level city	1.39 (1.12–1.72)	0.003	1.38 (1.11–1.72)	0.004
Rank of kindergarten (Ref: Provincial/demonstration level)		< 0.001		< 0.001
City-county level	0.64 (0.54–0.77)	< 0.001	0.65 (0.54–0.77)	< 0.001
Unrated level	0.77 (0.63–0.95)	0.016	0.79 (0.64–0.98)	0.028
Type of kindergarten (Ref: Private)	0.84 (0.71–1.01)	0.056	0.85 (0.71–1.01)	0.065
Premature delivery (Ref: No)		0.162		0.110
Yes	1.05 (0.83–1.34)	0.675	1.08 (0.84–1.37)	0.552
Undisclosed	0.67 (0.44–1.03)	0.067	0.65 (0.42–1.00)	0.048
Basic medical insurance (Ref: Yes)		0.004		0.005
No	0.57 (0.38–0.84)	0.005	0.59 (0.39–0.87)	0.009
Undisclosed	0.70 (0.47–1.02)	0.066	0.66 (0.45–0.98)	0.040
Commercial medical insurance (Ref: Yes)		< 0.001		< 0.001
No	0.67 (0.57–0.79)	< 0.001	0.69 (0.58–0.81)	< 0.001
Undisclosed	0.76 (0.58–0.99)	0.043	0.80 (0.61–1.05)	0.113
The only child in the family (Ref: No)	1.16 (1.00–1.35)	0.047	1.12 (0.96–1.30)	0.151
Complete vaccination for target-age children in immunization program (Ref: No)	1.84 (1.32–2.57)	< 0.001	1.73 (1.24–2.42)	0.001
Caregivers’ characteristics				
Primary caregiver (Ref: Other)		< 0.001		< 0.001
Mother	0.66 (0.54–0.82)	< 0.001	0.65 (0.53–0.80)	< 0.001
Father	0.73 (0.54–0.99)	0.045	0.75 (0.55–1.02)	0.070
Education level (Ref: Middle school and below)		< 0.001		< 0.001
Senior high school/technical school	1.43 (1.17–1.74)	< 0.001	1.40 (1.14–1.72)	0.001
College/associate’s degree	1.70 (1.39–2.09)	< 0.001	1.66 (1.34–2.04)	< 0.001
Bachelor’s degree and above	4.08 (2.30–7.25)	< 0.001	3.68 (2.04–6.63)	< 0.001
Does not know/unsure	0.88 (0.57–1.35)	0.547	0.89 (0.58–1.37)	0.585
Employment status (Ref: Employed)		0.045		0.036
Unemployed	0.79 (0.66–0.96)	0.017	0.78 (0.64–0.95)	0.012
Undisclosed	0.85 (0.63–1.16)	0.308	0.87 (0.63–1.20)	0.390
Annual household income (CNY) (Ref: < 30,000)		0.003		0.002
30,000–49,999	0.91 (0.66–1.25)	0.555	0.89 (0.64–1.24)	0.499
50,000–99,999	1.10 (0.82–1.49)	0.527	1.03 (0.76–1.40)	0.855
100,000–299,999	1.06 (0.80–1.41)	0.667	1.00 (0.75–1.33)	1.000
≥ 300,000	2.26 (1.47–3.47)	< 0.001	2.28 (1.47–3.55)	< 0.001
Undisclosed	1.09 (0.86–1.38)	0.478	1.05 (0.83–1.34)	0.668
Confidence				
Concern about vaccine safety (Ref: No)			0.58 (0.50–0.68)	< 0.001
Concern about vaccine efficacy (Ref: No)			1.53 (1.30–1.79)	< 0.001
Depend on doctors’ vaccination advice (Ref: No)			1.07 (0.92–1.25)	0.363
Concern about vaccine manufacturer (domestic or imported) (Ref: No)			2.05 (1.69–2.49)	< 0.001
Convenience				
Concern about vaccine price (Ref: No)			0.73 (0.60–0.88)	0.001
Concern about immunization schedule (dose and office visit) (Ref: No)			1.26 (1.04–1.54)	0.020
Concern about convenience of vaccination (distance, time, and appointment) (Ref: No)			1.14 (0.92–1.41)	0.237
Complacency				
Caregiver-reported health status for children (Ref: Fair/poor)			1.13 (0.93–1.36)	0.217

Adjusted odds ratio and 95% confidence intervals are presented

a*OR* Adjusted odd ratio; *CI* Confidential interval, *CNY* Chinese Yuan

^a^Model 2 was adjusted for socioeconomic and demographic characteristics. ^b^ represents the location of registered residency of the child (urban or rural)

### Assessing non-response bias

The analyses showed that responders (*n* = 4818) were comparable to non-responders (*n* = 3660) with regard to gender and age. Responders, however, were significantly more often of Han ethnicity and were county or autonomous county-, provincial-, or demonstration-level kindergarteners, or public kindergarteners compared to non-responders (*P* < 0.001) (Table 3). The subgroup analysis results for variables with differences are displayed in Additional file 1: Tables S1–S4.

**Table 3:** Demographics of responders and non-responders

Variable	Total (*N* = 8478)	Responder (*n* = 4818)	Non-responder (*n* = 3660)	*P*-value
Gender				0.960
Male	4651 (54.9)	2642 (54.8)	2009 (54.9)	
Female	3827 (45.1)	2176 (45.2)	1651 (45.1)	
Age (years)				0.813
≤ 3	6367 (75.1)	3623 (75.2)	2744 (75.0)	
4–5	2111 (24.9)	1195 (24.8)	916 (25.0)	
Ethnic group				< 0.001
Han	6555 (77.3)	3874 (80.4)	2681 (73.3)	
Minority	1923 (22.7)	944 (19.6)	979 (26.7)	
Administrative division				< 0.001
Municipal district	3360 (39.6)	1882 (39.1)	1478 (40.4)	
County-level city	2187 (25.8)	956 (19.8)	1231 (33.6)	
County/autonomous county	2931 (34.6)	1980 (41.1)	951 (26.0)	
Rank of kindergarten				< 0.001
Provincial/demonstration level	2528 (29.8)	1649 (34.2)	879 (24.0)	
City-county level	4500 (53.1)	2376 (49.3)	2124 (58.0)	
Unrated level	1450 (17.1)	793 (16.5)	657 (18.0)	
Type of kindergarten				< 0.001
Public	5493 (64.8)	3425 (71.1)	2068 (56.5)	
Private	2985 (35.2)	1393 (28.9)	1592 (43.5)	

Values are shown as *n* (%). *P*-values were derived from Chi-square tests

### Factors associated with DTaP-IPV/Hib vaccination in urban and rural areas

**Fig. 3:**
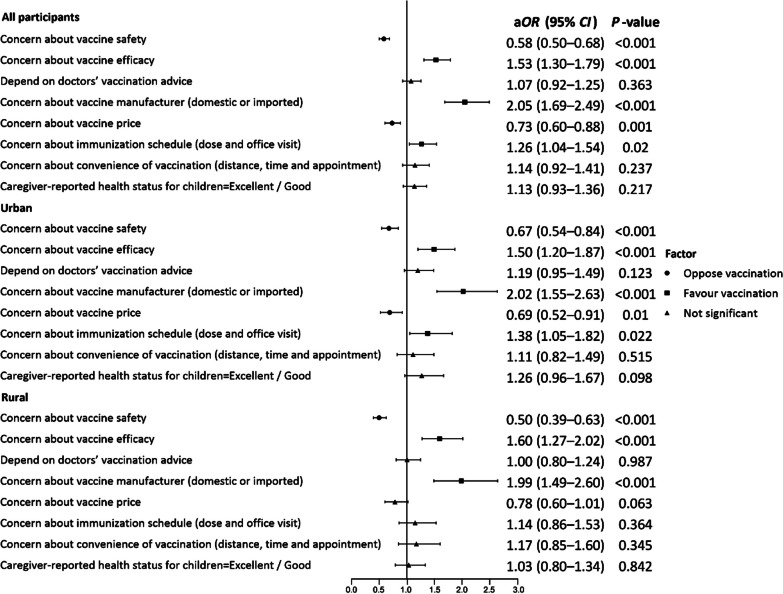
Factors associated with DTaP-IPV/Hib vaccination in urban and rural areas. We adjusted the socioeconomic and demographic characteristics of respondents. a*OR* adjusted odds ratio; *CI* confidence interval

In China, *hukou* represents the location of the registered residency of the children, which is approximately equal to the living residence. Our results showed that caregivers in both urban and rural groups are concerned about vaccine safety, efficacy, and the manufacturer (*P* < 0.001). Disparities were observed, however, in terms of the convenience dimension related to vaccine price and immunization schedule. Specifically, the urban group exhibited greater concerns regarding vaccine price (*P* = 0.010) and adherence to the immunization schedule (*P* = 0.022) in terms of vaccination against DTaP-IPV/Hib (Fig. 3).

## Discussion

The vaccination of children, the main target population, can have far-reaching effects on general health and well-being, cognitive development, and economic productivity. More than 70 vaccines are available for use, and many more are expected to protect against multiple diseases, which will further increase the number of injections and office visits [8, 26]. Complex immunization schedules can result in missed or delayed dosing, especially for children under 2 years old. Thus, it is essential to develop combination vaccines to simplify the vaccine schedule. Although the DTaP-IPV/Hib vaccine is the highest degree of combination vaccines available in China, the coverage rates of this vaccine are still low. It is, thus, critical to explore the factors that affect the vaccination rate of DTaP-IPV/Hib. To our knowledge, this is the first large sample investigation of the immunization status and the influencing factors of the DTap-IPV/Hib vaccine in Hainan Province, which includes more than ten million permanent residents. Our findings show that the cumulative coverage rates of the DTap-IPV/Hib vaccine from 1 to 4 doses were 24.4%, 20.7%, 18.5%, and 16.0%, respectively, in Hainan Province, which was higher than other areas in China [20, 21].

Consistent with other studies, Voo et al. found that caregivers with higher economic and cultural levels are more likely to vaccinate their children against DTap-IPV/Hib [27]. This could be explained by research that shows that caregivers with higher economic and cultural levels are inclined to accurately process the evidence regarding vaccination and to have access to more healthcare resources, such as choosing self-paid vaccines [28]. The cost of an imported DTaP-IPV/Hib vaccine per fully immunized child is estimated to be 2488 Chinese Yuan (CNY), and it is paid out-of-pocket, without any subsidy or insurance coverage. This higher cost may impose a financial burden on families with a lower income, potentially limiting their access to the vaccine and reducing the likelihood of full compliance and completion (Additional file 1: Table S5). Previous research in China and Japan has found that a subsidy would reduce the out-of-pocket price and increase the coverage of vaccination [29, 30].

Currently, the advancement of combined vaccines in China is impeded by numerous technical challenges. These include the absence of the crucial component IPV in the market and the presence of thiomersal in the co-purification process utilized for manufacturing DTaP vaccines, which can adversely affect the immunogenicity of the IPV antigen [31]. To address these concerns, the government should not only develop innovative vaccine pricing mechanisms and increase financing options but also provide an incentive for domestic manufacturers to research and develop DTap-IPV/Hib vaccines. Moreover, region-specific strategies should be developed based on their disease burden and fiscal capacity [32].

Our findings reveal that the efficacy and safety of the DTap-IPV/Hib vaccine have played a significant role in influencing its uptake within the general population. Studies in five countries in South America have revealed that safety and efficacy were the two most important factors for caregivers to decide whether to vaccinate their children [33]. Accurate information about vaccines is vital for caregivers, as they often lack a complete understanding of how vaccines function and struggle to make well-informed decisions about vaccination. Research conducted by Boerner et al. has shown that insufficient information about vaccination or conflicting information from various sources can decrease an individual’s willingness to vaccinate [34]. Therefore, it is of the utmost importance to communicate information about vaccines in a clear and easily comprehensible manner to overcome barriers to vaccination [35]. Healthcare providers play a crucial role as trusted sources of information for caregivers, enabling them to enhance their understanding and awareness. France’s implementation experience highlights the effectiveness of health education campaigns led by reputable medical institutions. These campaigns serve as valuable strategies to provide credible and reliable information about the safety and efficacy of vaccines. The ultimate goal is to empower individuals to make informed decisions regarding vaccination and ensure accessible and comprehensive vaccine information and knowledge [36].

The findings of this study demonstrated a notable disparity in vaccination rates between urban (35.7%) and rural populations (16.6%). In addition, the study revealed that caregivers who expressed concerns about the immunization schedule were more inclined to vaccinate their children against DTaP-IPV/Hib, particularly among those who resided in urban areas. As a combination vaccine, the DTap-IPV/Hib vaccine could simplify the immunization schedule and reduce the total number of required office visits [37]. Our preliminary research found that the Hib vaccination coverage rate in Hainan Province is 39.7%. Among children who received the Hib vaccine, 61.5% opted for direct vaccination with the DTap-IPV/Hib vaccine (data have not been published). Due to conflicts between routine vaccination times and parents’ working hours, parents in urban areas prefer to pay higher fees to buy time. Time loss related to the number of office visits may prevent parents from completing the immunization schedule on time and result in missed or delayed dosing. Pellissier et al. provided evidence that reducing the number of office visits can lead to time savings and potentially lower indirect costs associated with parental work loss [15]. Overall, although combination vaccines may cost slightly more than the total cost of their component vaccines, the benefits of vaccination timeliness and compliance and a simplified schedule may outweigh the cost.

This study has several limitations. First, there is a non-response bias in the study results due to the lower response rate. Responders and non-responders may differ in their vaccination status. Thus, we collected the available data to conduct an analysis of non-responders and then conducted a subgroup analysis. Second, the confirmation of vaccination status was based on the caregivers’ self‐reports, which rely on memory rather than medical records. Hence, the information may not accurately reflect the DTaP-IPV/Hib vaccine coverage rate and may be subject to recall bias. Because the newly enrolled children are required to provide vaccination records upon admission to kindergarten in September, however, the probability is less that their parents do not remember or are uncertain about the DTaP-IPV/Hib vaccination status. Third, the sample was selected from one geographic area. The specific context of Hainan Province, which might not represent the whole population in China, could limit the generalizability of the findings. Further research should be undertaken to extend the scope to widely evaluate the vaccination rate and influencing factors in China. Despite the above limitations, this study provides important evidence by which to evaluate the vaccination status and popularization proposals of the DTaP-IPV/Hib vaccine in China.

## Conclusions

Our study provides important evidence of the prevalence and determining factors of the DTaP-IPV/Hib vaccination in Hainan Province, China. The coverage rate of the DTaP-IPV/Hib vaccine in Hainan Province remains at a low level but is slightly higher than that found in previous studies conducted in China. Caregivers may be hesitant to vaccinate their children against DTaP-IPV/Hib due to concerns about the vaccine’s safety and price. Thus, more effective health education campaigns should be conducted to publicize and promote access to DTaP-IPV/Hib vaccine knowledge and awareness. Further, the government should provide an incentive for domestic manufacturers to research and develop DTap-IPV/Hib vaccines as well as provide innovative vaccine pricing mechanisms and increase financing options to address the cost concern.

### Supplementary Information

**Additional file 1:** **Table S1**. Multivariate analysis of the potential factors that influence the DTaP-IPV/Hib vaccination by ethnicity. **Table S2**. Multivariate analysis of the potential factors that influence the DTaP-IPV/Hib vaccination by kindergarten rank. **Table S3**. Multivariate analysis of the potential factors that influence the DTaP-IPV/Hib vaccination by type of kindergarten. **Table S4**. Multivariate analysis of the potential factors that influence the DTaP-IPV/Hib vaccination by county-level administrative region. **Table S5**. Multivariate analysis of the potential factors that influence the full-course DTaP-IPV/Hib vaccination (*N* = 1174).

## Data Availability

The datasets used and/or analyzed during the current study are available from the corresponding author on reasonable request.

## Abbreviations

ECD: Early child development
US dollars: USD
WHO: World Health Organization
VPDs: Vaccine-preventable diseases
NIP: National Immunization Program
DTaP-IPV/Hib: Diphtheria-tetanus-acellular pertussis inactivated poliomyelitis and *Haemophilus influenzae* type B
HFTP: Hainan Free Trade Port
PSUs: Primary sampling units
SSUs: Secondary sampling units
a*OR*: Adjusted odd ratio
*CI*: Confidential interval
CNY: Chinese Yuan
