# Supplementary material for: Prevalence and factors associated with pentavalent vaccination: a cross-sectional study in Southern China
Source: Infect Dis Poverty. 2023 Sep 15;12:84. doi: 10.1186/s40249-023-01134-8 (PMC10502987; doi:10.1186/s40249-023-01134-8)
Supplement: Supplementary file 1 — Additional file 1: Table S1. Multivariate analysis of the potential factors that influence the DTaP-IPV/Hib vaccination by ethnicity. Table S2. Multivariate analysis of the potential factors that influence the DTaP-IPV/Hib vaccination by kindergarten rank. Table S3. Multivariate analysis of the potential factors that influence the DTaP-IPV/Hib vaccination by type of kindergarten. Table S4. Multivariate analysis of the potential factors that influence the DTaP-IPV/Hib vaccination by county-level administrative region. Table S5. Multivariate analysis of the potential factors that influence the full-course DTaP-IPV/Hib vaccination (N = 1174). [file 40249_2023_1134_MOESM1_ESM.docx]

**Additional file**

| Table S1. Multivariate analysis of the potential factors that influence the DTaP-IPV/Hib vaccination by ethnic. | | | | |
| --- | --- | --- | --- | --- |
| **Variable** | **Han population (*n* = 3874)** | | **Minority population (*n* = 944)** | |
|  | **a*OR* (95% *CI*)** | ***P* value** | **a*OR* (95% *CI*)** | ***P* value** |
| **Children’s characteristics** |  |  |  |  |
| Gender (Ref: Female) | 0.989(0.84–1.15) | 0.872 | 0.95(0.63–1.45) | 0.823 |
| Age (Ref: ≤ 3 years ) | 0.94(0.78–1.14) | 0.547 | 1.03(0.654–1.63) | 0.892 |
| HuKou^a^ (Ref: Rural) | 1.33(1.12–1.59) | 0.001 | 1.87(1.13–3.12) | 0.016 |
| County-level administrative region (Ref: County/ Autonomous county) |  | <0.001 |  | 0.082 |
| Municipal district | 2.08(1.70–2.55) | <0.001 | 1.55(0.98–2.47) | 0.064 |
| County-level city | 1.39(1.10–1.75) | 0.005 | 2.05(0.83–5.07) | 0.120 |
| Rank of kindergartens (Ref: Provincial/Demonstration level) |  | <0.001 |  | 0.183 |
| City-County level | 0.64(0.53–0.78) | <0.001 | 0.58(0.33–1.04) | 0.065 |
| Unrated level | 0.77(0.61–0.97) | 0.023 | 0.72(0.35–1.50) | 0.382 |
| Type of kindergartens (Ref: Private) | 0.93(0.77–1.13) | 0.461 | 0.47(0.27–0.83) | 0.009 |
| Premature delivery (Ref: No) |  | 0.054 |  | 0.092 |
| Yes | 0.99(0.75–1.30) | 0.926 | 1.85(1.04–3.27) | 0.036 |
| Undisclosed | 0.55(0.34–0.89) | 0.016 | 1.50(0.56–3.99) | 0.418 |
| Basic medical insurance (Ref: Yes) |  | 0.001 |  | 0.743 |
| No | 0.53(0.35–0.82) | 0.004 | 0.94(0.28–3.15) | 0.924 |
| Undisclosed | 0.56(0.36–0.89) | 0.013 | 1.38(0.60–3.17) | 0.449 |
| Commercial medical insurance (Ref: Yes) |  | <0.001 |  | 0.724 |
| No | 0.68(0.57–0.81) | <0.001 | 0.82(0.49–1.37) | 0.453 |
| Undisclosed | 0.80(0.59–1.08) | 0.138 | 0.94(0.44–2.00) | 0.876 |
| The only child in the family (Ref: No) | 1.20(1.02–1.42) | 0.030 | 0.68(0.44–1.05) | 0.079 |
| Complete the vaccination for the target age children in immunization programme of Hainan Province (Ref: No) | 1.68(1.17–2.41) | 0.005 | 2.36(0.87–6.40) | 0.092 |
| **Caregivers’ characteristics** |  |  |  |  |
| Primary caregiver (Ref: Other) |  | 0.001 |  | 0.722 |
| Mother | 0.65(0.52–0.82) | <0.001 | 0.84(0.44–1.60) | 0.601 |
| Father | 0.79(0.56–1.1) | 0.161 | 0.68(0.26–1.76) | 0.421 |
| Education level (Ref: Middle school and below) |  | <0.001 |  | 0.062 |
| Senior high school/ Technical school | 1.36(1.09–1.69) | 0.007 | 1.56(0.88–2.76) | 0.130 |
| College/ Associate degree | 1.55(1.24–1.95) | <0.001 | 2.02(1.09–3.73) | 0.025 |
| Bachelor’s degree and above | 3.18(1.72–5.85) | <0.001 | 17.63(1.54–201.76) | 0.021 |
| Not sure | 0.76(0.47–1.23) | 0.26 | 1.82(0.62–5.36) | 0.278 |
| Employment status (Ref: Employed) |  | 0.029 |  | 0.786 |
| Unemployed | 0.76(0.62–0.94) | 0.012 | 0.88(0.52–1.49) | 0.632 |
| Undisclosed | 0.81(0.58–1.15) | 0.243 | 1.19(0.53–2.70) | 0.674 |
| Annual household income (CNY) (Ref: < 30,000) |  | <0.001 |  | 0.467 |
| 30,000–49,999 | 0.89(0.62–1.27) | 0.509 | 0.74(0.32–1.71) | 0.483 |
| 50,000–99,999 | 0.92(0.66–1.29) | 0.641 | 1.40(0.66–2.98) | 0.386 |
| 100,000–299,999 | 0.91(0.66–1.24) | 0.533 | 1.61(0.75–3.47) | 0.223 |
| ≥ 300,000 | 2.42(1.51–3.87) | <0.001 | 0.70(0.11–4.26) | 0.696 |
| Undisclosed | 1.02(0.78–1.34) | 0.884 | 0.94(0.53–1.67) | 0.838 |
| Adjusted odds ratio and 95% confidence intervals were presented.  a Hukou represent the location of registered residency of the child. Here we divided it into urban or rural.  a*OR*: adjusted odd ratio; *CI*: Confidential interval; CNY: Chinese Yuan. | | | | |

| Table S2. Multivariate analysis of the potential factors that influence the DTaP-IPV/Hib vaccination by rank of kindergartens. | | | | | | |
| --- | --- | --- | --- | --- | --- | --- |
| **Variable** | **Provincial/ Demonstration level (*n* = 1649)** | | **City-County level (*n* = 2376)** | | **Unrated level (*n* = 793)** | |
|  | **a*OR* (95% *CI*)** | ***P* value** | **a*OR* (95% *CI*)** | ***P* value** | **a*OR* (95% *CI*)** | ***P* value** |
| **Children’s characteristics** |  |  |  |  |  |  |
| Gender (Ref: Female) | 0.93(0.73–1.18) | 0.555 | 1.08(0.86–1.35) | 0.520 | 0.98(0.68–1.41) | 0.904 |
| Age (Ref: ≤ 3 years) | 0.93(0.71–1.22) | 0.594 | 0.84(0.64–1.10) | 0.213 | 1.35(0.87–2.11) | 0.179 |
| HuKou^a^ (Ref: Rural) | 1.80(1.36–2.39) | <0.001 | 1.13(0.88–1.456) | 0.334 | 1.63(1.07–2.50) | 0.024 |
| Ethnic group (Ref: Minority) | 1.44(1.01–2.06) | 0.043 | 1.36(0.97–1.91) | 0.075 | 1.36(0.73–2.52) | 0.331 |
| County-level administrative region (Ref: County/Autonomous county) |  | <0.001 |  | <0.001 |  | 0.001 |
| Municipal district | 2.69(1.92–3.77) | <0.001 | 1.46(1.13–1.89) | 0.004 | 2.05(1.22–3.42) | 0.006 |
| County-level city | 1.26(0.85–1.87) | 0.253 | 1.95(1.44–2.66) | <0.001 | 0.99(0.54–1.82) | 0.969 |
| Type of kindergartens (Ref: Private) | 0.61(0.39–0.95) | 0.027 | 0.90(0.72–1.13) | 0.357 | 0.93(0.58–1.47) | 0.744 |
| Premature delivery (Ref: No) |  | 0.392 |  | 0.593 |  | 0.540 |
| Yes | 1.15(0.76–1.74) | 0.524 | 1.07(0.74–1.55) | 0.714 | 1.00(0.55–1.83) | 0.999 |
| Undisclosed | 0.66(0.32–1.33) | 0.241 | 0.73(0.38–1.41) | 0.354 | 0.55(0.19–1.58) | 0.267 |
| Basic medical insurance (Ref: Yes) |  | 0.022 |  | 0.143 |  | 0.572 |
| No | 0.37(0.17–0.79) | 0.010 | 0.69(0.40–1.18) | 0.175 | 0.83(0.31–2.23) | 0.717 |
| Undisclosed | 0.68(0.34–1.37) | 0.280 | 0.66(0.38–1.14) | 0.138 | 0.59(0.21–1.64) | 0.313 |
| Commercial medical insurance (Ref: Yes) |  | 0.105 |  | 0.001 |  | 0.346 |
| No | 0.76(0.58–0.98) | 0.034 | 0.60(0.46–0.78) | <0.001 | 0.75(0.48–1.15) | 0.186 |
| Undisclosed | 0.86(0.53–1.38) | 0.522 | 0.65(0.43–0.98) | 0.040 | 0.96(0.50–1.85) | 0.894 |
| The only child in the family (Ref: No) | 1.04(0.80–1.33) | 0.788 | 1.20(0.95–1.5) | 0.122 | 1.02(0.69–1.52) | 0.909 |
| Complete the vaccination for the target age children in immunization programme of Hainan Province (Ref: No) | 1.49(0.86–2.56) | 0.152 | 2.51(1.38–4.56) | 0.002 | 1.22(0.60–2.48) | 0.578 |
| **Caregivers’ characteristics** |  |  |  |  |  |  |
| Primary caregiver (Ref: Other) |  | 0.013 |  | 0.151 |  | 0.053 |
| Mother | 0.60(0.42–0.86) | 0.005 | 0.73(0.53–1.01) | 0.060 | 0.53(0.31–0.88) | 0.015 |
| Father | 0.81(0.48–1.36) | 0.423 | 0.85(0.53–1.38) | 0.510 | 0.59(0.28–1.25) | 0.166 |
| Education level (Ref: Middle school and below) |  | 0.009 |  | <0.001 |  | 0.701 |
| Senior high school/ Technical school | 1.20(0.83–1.74) | 0.337 | 1.63(1.23–2.16) | 0.001 | 1.20(0.71–2.02) | 0.495 |
| College/ Associate degree | 1.86(1.30–2.66) | 0.001 | 1.74(1.26–2.39) | 0.001 | 1.13(0.66–1.93) | 0.656 |
| Bachelor’s degree and above | 2.34(1.08–5.07) | 0.031 | 15.17(3.97–58.05) | <0.001 | 1.65(0.29–9.36) | 0.571 |
| Not sure | 1.15(0.55–2.38) | 0.715 | 0.93(0.50–1.73) | 0.811 | 0.48(0.13–1.77) | 0.270 |
| Employment status (Ref: Employed) |  | 0.010 |  | 0.291 |  | 0.225 |
| Unemployed | 0.62(0.44–0.88) | 0.007 | 0.88(0.66–1.16) | 0.351 | 0.74(0.46–1.20) | 0.219 |
| Undisclosed | 0.58(0.30–1.13) | 0.108 | 1.27(0.83–1.93) | 0.271 | 0.58(0.26–1.26) | 0.169 |
| Annual household income (CNY) (Ref: < 30,000) |  | 0.495 |  | 0.110 |  | 0.055 |
| 30,000–49,999 | 1.05(0.57–1.93) | 0.877 | 0.77(0.49–1.22) | 0.262 | 1.23(0.54–2.78) | 0.625 |
| 50,000–99,999 | 1.04(0.60–1.79) | 0.904 | 1.21(0.80–1.83) | 0.375 | 0.68(0.29–1.60) | 0.374 |
| 100,000–299,999 | 0.96(0.59–1.59) | 0.885 | 0.88(0.57–1.35) | 0.556 | 1.43(0.69–2.98) | 0.341 |
| ≥ 300,000 | 1.69(0.87–3.31) | 0.124 | 2.34(1.00–5.44) | 0.049 | 4.10(1.35–12.39) | 0.013 |
| Undisclosed | 0.99(0.63–1.57) | 0.973 | 0.95(0.68–1.31) | 0.738 | 1.36(0.73–2.55) | 0.332 |
| Adjusted odds ratio and 95% confidence intervals were presented.  a Hukou represent the location of registered residency of the child. Here we divided it into urban or rural.  a*OR*: adjusted odd ratio; *CI*: Confidential interval; CNY: Chinese Yuan. | | | | | | |

| Table S3. Multivariate analysis of the potential factors that influence the DTaP-IPV/Hib vaccination by type of kindergartens. | | | | |
| --- | --- | --- | --- | --- |
| **Variable** | **Public kindergarten (*n* = 3425)** | | **Private kindergarten (*n* = 1393)** | |
|  | **a*OR* (95% *CI*)** | ***P* value** | **a*OR* (95% *CI*)** | ***P* value** |
| **Children’s characteristics** |  |  |  |  |
| Gender (Ref: Female) | 0.95(0.80–1.12) | 0.525 | 1.10(0.83–1.46) | 0.520 |
| Age (Ref: ≤ 3 years) | 0.89(0.73–1.09) | 0.265 | 0.99(0.70–1.39) | 0.940 |
| HuKou^a^ (Ref: Rural) | 1.53(1.26–1.87) | <0.001 | 1.20(0.88–1.65) | 0.252 |
| Ethnic group (Ref: Minority) | 1.76(1.34–2.31) | <0.001 | 0.95(0.64–1.42) | 0.804 |
| County-level administrative region (Ref: County/Autonomous county) |  | <0.001 |  | 0.020 |
| Municipal district | 2.36(1.88–2.95) | <0.001 | 1.19(0.84–1.67) | 0.326 |
| County-level city | 1.28(0.98–1.68) | 0.071 | 1.78(1.18–2.69) | 0.006 |
| Rank of kindergartens (Ref: Provincial/ Demonstration level) |  | <0.001 |  | 0.065 |
| City-County level | 0.66(0.54–0.81) | <0.001 | 0.58(0.36–0.94) | 0.025 |
| Unrated level | 0.77(0.61–0.98) | 0.030 | 0.70(0.39–1.28) | 0.247 |
| Premature delivery (Ref: No) |  | 0.061 |  | 0.560 |
| Yes | 1.2(0.90–1.60) | 0.212 | 0.78(0.48–1.28) | 0.331 |
| Undisclosed | 0.60(0.36–1.01) | 0.053 | 0.81(0.36–1.80) | 0.598 |
| Basic medical insurance (Ref: Yes) |  | 0.003 |  | 0.684 |
| No | 0.48(0.29–0.78) | 0.003 | 0.87(0.42–1.77) | 0.693 |
| Undisclosed | 0.62(0.38–1.03) | 0.063 | 0.76(0.39–1.49) | 0.427 |
| Commercial medical insurance (Ref: Yes) |  | 0.036 |  | <0.001 |
| No | 0.77(0.64–0.94) | 0.010 | 0.45(0.32–0.63) | <0.001 |
| Undisclosed | 0.84(0.60–1.18) | 0.311 | 0.65(0.40–1.05) | 0.079 |
| The only child in the family (Ref: No) | 1.17(0.97–1.40) | 0.099 | 0.97(0.72–1.30) | 0.820 |
| Complete the vaccination for the target age children in immunization programme of Hainan Province (Ref: No) | 1.68(1.13–2.51) | 0.011 | 2.10(1.09–4.04) | 0.026 |
| **Caregivers’ characteristics** |  |  |  |  |
| Primary caregiver (Ref: Other) |  | 0.017 |  | 0.049 |
| Mother | 0.70(0.54–0.90) | 0.005 | 0.61(0.41–0.91) | 0.016 |
| Father | 0.80(0.56–1.15) | 0.233 | 0.76(0.39–1.48) | 0.420 |
| Education level (Ref: Middle school and below) |  | 0.002 |  | 0.004 |
| Senior high school/ Technical school | 1.26(0.98–1.61) | 0.074 | 1.65(1.14–2.38) | 0.008 |
| College/ Associate degree | 1.46(1.14–1.88) | 0.003 | 1.91(1.27–2.86) | 0.002 |
| Bachelor’s degree and above | 2.72(1.42–5.22) | 0.003 | 7.26(1.59–33.18) | 0.011 |
| Not sure | 0.73(0.41–1.28) | 0.265 | 1.09(0.54–2.23) | 0.807 |
| Employment status (Ref: Employed) |  | 0.007 |  | 0.708 |
| Unemployed | 0.68(0.53–0.86) | 0.002 | 0.98(0.69–1.38) | 0.895 |
| Undisclosed | 0.94(0.64–1.37) | 0.734 | 0.77(0.42–1.42) | 0.406 |
| Annual household income (CNY) (Ref: < 30,000) |  | 0.013 |  | 0.334 |
| 30,000–49,999 | 0.80(0.54–1.20) | 0.289 | 1.09(0.61–1.95) | 0.782 |
| 50,000–99,999 | 0.91(0.63–1.31) | 0.621 | 1.35(0.75–2.41) | 0.318 |
| 100,000–299,999 | 0.86(0.61–1.20) | 0.367 | 1.25(0.72–2.19) | 0.430 |
| ≥ 300,000 | 1.92(1.16–3.16) | 0.011 | 3.13(1.08–9.08) | 0.036 |
| Undisclosed | 0.92(0.68–1.24) | 0.583 | 1.37(0.89–2.10) | 0.150 |
| Adjusted odds ratio and 95% confidence intervals were presented.  a Hukou represent the location of registered residency of the child. Here we divided it into urban or rural.  a*OR*: adjusted odd ratio; *CI*: Confidential interval; CNY: Chinese Yuan. | | | | |

| Table S4. Multivariate analysis of the potential factors that influence the DTaP-IPV/Hib vaccination by county-level administrative region. | | | | | | |
| --- | --- | --- | --- | --- | --- | --- |
| **Variable** | **Municipal district (*n* = 1882)** | | **County-level city (*n* = 956)** | | **County/Autonomous county (*n* = 1980)** | |
|  | **a*OR* (95% *CI*)** | ***P* value** | **a*OR* (95% *CI*)** | ***P* value** | **a*OR* (95% *CI*)** | ***P* value** |
| **Children’s characteristics** |  |  |  |  |  |  |
| Gender (Ref: Female) | 0.93(0.75–1.15) | 0.524 | 1.00(0.73–1.37) | 0.973 | 1.08(0.82–1.42) | 0.574 |
| Age (Ref: ≤ 3 years) | 0.92(0.72–1.19) | 0.536 | 0.97(0.66–1.43) | 0.883 | 0.99(0.72–1.36) | 0.944 |
| HuKou^a^ (Ref: Rural) | 1.42(1.11–1.83) | 0.006 | 1.29(0.90–1.85) | 0.159 | 1.52(1.12–2.07) | 0.008 |
| Ethnic group (Ref: Minority) | 1.86(1.27–2.75) | 0.002 | 0.83(0.36–1.94) | 0.673 | 1.26(0.93–1.70) | 0.138 |
| Rank of kindergartens (Ref: Provincial/ Demonstration level) |  | <0.001 |  | 0.377 |  | 0.237 |
| City-County level | 0.49(0.38–0.64) | <0.001 | 1.08(0.74–1.58) | 0.689 | 0.77(0.53–1.11) | 0.159 |
| Unrated level | 0.74(0.55–1.00) | 0.050 | 0.76(0.48–1.21) | 0.250 | 0.98(0.61–1.58) | 0.942 |
| Type of kindergartens (Ref: Private) | 1.17(0.87–1.55) | 0.300 | 0.63(0.44–0.92) | 0.016 | 0.71(0.51–0.97) | 0.034 |
| Premature delivery (Ref: No) |  | 0.277 |  | 0.902 |  | 0.373 |
| Yes | 1.16(0.81–1.66) | 0.423 | 1.06(0.58–1.91) | 0.858 | 1.13(0.74–1.72) | 0.580 |
| Undisclosed | 0.66(0.35–1.22) | 0.182 | 0.82(0.32–2.12) | 0.684 | 0.59(0.26–1.34) | 0.210 |
| Basic medical insurance (Ref: Yes) |  | 0.031 |  | 0.016 |  | 0.943 |
| No | 0.51(0.28–0.90) | 0.020 | 0.44(0.18–1.08) | 0.072 | 0.93(0.46–1.867) | 0.832 |
| Undisclosed | 0.66(0.36–1.22) | 0.187 | 0.28(0.10–0.83) | 0.021 | 1.08(0.60–1.97) | 0.796 |
| Commercial medical insurance (Ref: Yes) |  | 0.001 |  | 0.288 |  | 0.207 |
| No | 0.64(0.51–0.81) | <0.001 | 0.75(0.51–1.09) | 0.132 | 0.73(0.52–1.03) | 0.077 |
| Undisclosed | 0.78(0.51–1.20) | 0.257 | 0.91(0.49–1.68) | 0.765 | 0.76(0.46–1.24) | 0.265 |
| The only child in the family (Ref: No) | 1.27(1.02–1.59) | 0.035 | 0.94(0.67–1.32) | 0.718 | 1.01(0.75–1.36) | 0.952 |
| Complete the vaccination for the target age children in immunization programme of Hainan Province (Ref: No) | 1.69(1.02–2.80) | 0.041 | 2.13(1.00–4.55) | 0.051 | 1.80(0.99–3.24) | 0.053 |
| **Caregivers’ characteristics** |  |  |  |  |  |  |
| Primary caregiver (Ref: Other) |  | 0.151 |  | 0.245 |  | 0.005 |
| Mother | 0.74(0.54–1.01) | 0.054 | 0.65(0.40–1.08) | 0.094 | 0.58(0.40–0.85) | 0.005 |
| Father | 0.74(0.47–1.18) | 0.209 | 0.71(0.35–1.46) | 0.353 | 0.95(0.55–1.65) | 0.855 |
| Education level (Ref: Middle school and below) |  | 0.002 |  | 0.091 |  | 0.022 |
| Senior high school/ Technical school | 1.53(1.10–2.12) | 0.011 | 1.20(0.77–1.89) | 0.416 | 1.46(1.04–2.05) | 0.028 |
| College/ Associate degree | 1.68(1.21–2.31) | 0.002 | 1.79(1.14–2.80) | 0.012 | 1.56(1.05–2.32) | 0.030 |
| Bachelor’s degree and above | 3.56(1.77–7.15) | <0.001 | 2.26(0.43–11.86) | 0.334 | 12.23(0.95–157.29) | 0.055 |
| Not sure | 1.23(0.65–2.31) | 0.526 | 0.68(0.27–1.72) | 0.416 | 0.61(0.25–1.49) | 0.276 |
| Employment status (Ref: Employed) |  | 0.038 |  | 0.437 |  | 0.824 |
| Unemployed | 0.69(0.51–0.94) | 0.018 | 0.76(0.50–1.16) | 0.204 | 0.91(0.65–1.28) | 0.596 |
| Undisclosed | 0.74(0.45–1.20) | 0.221 | 0.88(0.45–1.71) | 0.700 | 1.07(0.62–1.85) | 0.812 |
| Annual household income (CNY) (Ref: < 30,000) |  | 0.243 |  | 0.020 |  | 0.129 |
| 30,000–49,999 | 0.95(0.51–1.78) | 0.873 | 0.56(0.28–1.09) | 0.088 | 1.03(0.63–1.67) | 0.905 |
| 50,000–99,999 | 1.05(0.62–1.77) | 0.866 | 0.52(0.25–1.07) | 0.077 | 1.55(0.96–2.50) | 0.074 |
| 100,000–299,999 | 1.07(0.66–1.74) | 0.778 | 0.64(0.35–1.18) | 0.152 | 1.12(0.65–1.93) | 0.677 |
| ≥ 300,000 | 1.88(1.02–3.48) | 0.044 | 2.77(0.90–8.55) | 0.077 | 3.99(1.20–13.32) | 0.024 |
| Undisclosed | 1.07(0.68–1.68) | 0.777 | 0.92(0.56–1.50) | 0.732 | 1.05(0.72–1.53) | 0.803 |
| Adjusted odds ratio and 95% confidence intervals were presented.  a Hukou represent the location of registered residency of the child. Here we divided it into urban or rural.  a*OR*: adjusted odd ratio; *CI*: Confidential interval; CNY: Chinese Yuan. | | | | | | |

| Table S5. Multivariate analysis of the potential factors that influence the full-course DTaP-IPV/Hib vaccination (*n* = 1174). | | |
| --- | --- | --- |
| **Variable** | **a*OR* (95% *CI*)** | ***P* value** |
| **Children’s characteristics** |  |  |
| Gender (Ref: Female) | 1.15(0.88–1.49) | 0.313 |
| Age (Ref: ≤ 3 years) | 1.03(0.75–1.41) | 0.847 |
| HuKou^a^ (Ref: Rural) | 1.17(0.87–1.58) | 0.305 |
| Ethnic group (Ref: Minority) | 0.94(0.61–1.40) | 0.765 |
| County–level administrative region (Ref: County/Autonomous county) |  | 0.037 |
| Municipal district | 1.47(1.04–2.08) | 0.031 |
| County-level city | 1.02(0.69–1.52) | 0.921 |
| Rank of kindergartens (Ref: Provincial/ Demonstration level) |  | 0.190 |
| City-County level | 1.04(0.75–1.46) | 0.810 |
| Unrated level | 0.74(0.51–1.08) | 0.119 |
| Type of kindergartens (Ref: Private) | 1.11(0.80–1.54) | 0.523 |
| Premature delivery (Ref: No) |  | 0.828 |
| Yes | 0.98(0.63–1.52) | 0.920 |
| Undisclosed | 0.78(0.35–1.74) | 0.541 |
| Basic medical insurance (Ref: Yes) |  | 0.305 |
| No | 0.56(0.27–1.17) | 0.124 |
| Undisclosed | 0.94(0.45–2.00) | 0.877 |
| Commercial medical insurance (Ref: Yes) |  | 0.240 |
| No | 0.79(0.59–1.07) | 0.125 |
| Undisclosed | 0.72(0.44–1.18) | 0.197 |
| The only child in the family (Ref: No) | 1.09(0.83–1.44) | 0.534 |
| Complete the vaccination for the target age children in immunization program of Hainan Province (Ref: No) | 1.62(0.84–3.13) | 0.153 |
| **Caregivers’ characteristics** |  |  |
| Primary caregiver (Ref: Other) |  | 0.081 |
| Mother | 0.85(0.59–1.23) | 0.396 |
| Father | 0.54(0.31–0.94) | 0.028 |
| Education level (Ref: Middle school and below) |  | 0.007 |
| Senior high school/ Technical school | 1.15(0.79–1.66) | 0.472 |
| College/ Associate degree | 1.73(1.17–2.54) | 0.006 |
| Bachelor’s degree and above | 2.30(0.92–5.75) | 0.076 |
| Not sure | 0.53(0.24–1.20) | 0.127 |
| Employment status (Ref: Employed) |  | 0.620 |
| Unemployed | 1.05(0.73–1.52) | 0.784 |
| Undisclosed | 0.77(0.43–1.37) | 0.371 |
| Annual household income (CNY) (Ref: < 30,000) |  | 0.006 |
| 30,000–49,999 | 2.03(1.10–3.76) | 0.024 |
| 50,000–99,999 | 1.75(1.00–3.07) | 0.051 |
| 100,000–299,999 | 2.82(1.66–4.79) | <0.001 |
| ≥ 300,000 | 2.80(1.35–5.81) | 0.006 |
| Undisclosed | 1.87(1.19–2.94) | 0.006 |
| **Confidence** | | |
| Concern about vaccine safety (Ref: No) | 0.91(0.68–1.22) | 0.527 |
| Concern about vaccine efficacy (Ref: No) | 1.51(1.12–2.03) | 0.006 |
| Depend on doctors’ vaccination advice (Ref: No) | 1.12(0.85–1.49) | 0.424 |
| Concern about vaccine manufacturer (domestic or imported) (Ref: No) | 0.91(0.66–1.26) | 0.576 |
| **Convenience** | | |
| Concern about vaccine price (Ref: No) | 0.62(0.44–0.87) | 0.006 |
| Concern about immunization schedule (dose and office visit) (Ref: No) | 1.22(0.86–1.73) | 0.260 |
| Concern about convenience of vaccination (distance, time and appointment) (Ref: No) | 0.98(0.67–1.43) | 0.912 |
| **Complacency** | | |
| Caregiver–reported health status for children (Ref: Fair/Poor) | 1.00(0.71–1.43) | 0.983 |
| Adjusted odds ratio and 95% confidence intervals were presented.  a Hukou represent the location of registered residency of the child. Here we divided it into urban or rural.  a*OR*: adjusted odd ratio; *CI*: Confidential interval; CNY: Chinese Yuan. | | |
